# Development and Experimental Validation of an Intelligent Camera Model for Automated Driving

**DOI:** 10.3390/s21227583

**Published:** 2021-11-15

**Authors:** Simon Genser, Stefan Muckenhuber, Selim Solmaz, Jakob Reckenzaun

**Affiliations:** 1 Virtual Vehicle Research GmbH, Inffeldgasse 21a, 8010 Graz, Austria; stefan.muckenhuber@v2c2.at (S.M.); selim.solmaz@v2c2.at (S.S.); jakob.reckenzaun@v2c2.at (J.R.); 2 Department of Geography and Regional Science, University of Graz, Heinrichstraße 36, 8010 Graz, Austria

**Keywords:** automotive perception sensors, sensor model, virtual testing, ADAS/AD function, automotive camera

## Abstract

The virtual testing and validation of advanced driver assistance system and automated driving (ADAS/AD) functions require efficient and realistic perception sensor models. In particular, the limitations and measurement errors of real perception sensors need to be simulated realistically in order to generate useful sensor data for the ADAS/AD function under test. In this paper, a novel sensor modeling approach for automotive perception sensors is introduced. The novel approach combines kernel density estimation with regression modeling and puts the main focus on the position measurement errors. The modeling approach is designed for any automotive perception sensor that provides position estimations at the object level. To demonstrate and evaluate the new approach, a common state-of-the-art automotive camera (Mobileye 630) was considered. Both sensor measurements (Mobileye position estimations) and ground-truth data (DGPS positions of all attending vehicles) were collected during a large measurement campaign on a Hungarian highway to support the development and experimental validation of the new approach. The quality of the model was tested and compared to reference measurements, leading to a pointwise position error of 9.60% in the lateral and 1.57% in the longitudinal direction. Additionally, the modeling of the natural scattering of the sensor model output was satisfying. In particular, the deviations of the position measurements were well modeled with this approach.

## 1. Introduction

According to the World Health Organization, more than 1.35 million people die in road traffic crashes each year, and up to 50 million are injured or become disabled. This makes road traffic crashes the leading cause of death among children and young adults between 5 y and 29 y [1]. Road traffic crashes are preventable, and advanced driver assistance system and automated driving (ADAS/AD) functions are meant to play an important role in improving safety both for vehicle passengers and vulnerable road users, such as pedestrians and cyclists [2,3]. ADAS/AD functions are furthermore developed to reduce emissions and congestion, increase driving comfort, and enable new transportation applications [4].

The higher the level of automation, the more benefits are expected. To classify the level of automation, SAE International defined six levels of driving automation [5]. Currently available vehicles provide up to SAE Level-2 automation, which is defined as “partial driving automation”. Examples of Level-2 systems are Tesla’s Autopilot, Nissan’s ProPILOT Assist, Cadillac’s Super Cruise, and Volvo’s Pilot Assist. “Partial driving automation” means that the system can take over lateral and longitudinal vehicle motion control, but the driver still has to monitor the driving environment and supervise the driving automation system. Hence, the driver is responsible for object and event detection and proper responses.

Systems capable of SAE Level-3 “conditional driving automation” take over object and event detection and responses. This implies that the driver can take his/her eyes off the road and is only required to intervene when the system requests this. The shift from Level-2 to Level-3 represents a major challenge, since the responsibility of the environment monitoring is transferred from the driver to the system. This requires a reliable and well-tested environment perception system. Diverse and redundant sensor types are needed to enable such a robust environment perception. A combination of cameras, radar, and LiDAR, is considered to eventually provide the necessary capabilities to fulfil the high demands of Level-3+ vehicles [6]. SAE Level-3 systems, such as the Mercedes DRIVE PILOT and Honda SENSING Elite system, are currently under test.

### 1.1. Role of Cameras in ADAS/AD Functions

The camera is a key sensor to achieve a reliable environment perception for ADAS/AD functions. Since the 1990s, all relevant AD demonstrators (as listed in Marti et al. [6]) included a camera in their perception systems; often, several cameras, sometimes even more than ten, have been used. Today, automotive camera systems are standard equipment in several middle- and high-class vehicles and support several Level-2 and Level-3 ADAS/AD functionalities, such as lane-keeping, adaptive cruise control, traffic jam assistance, as well as perception-oriented ADAS functions such as traffic sign and traffic light detection and recognition, object detection and classification, etc. [6]

Unlike radar and LiDAR, which are active sensors, the camera is a passive sensor. An external light source, either sunlight during the daytime or artificial light during the night, is required. The light from the external source is reflected by objects in the environment and partly forwarded in the direction of the camera. The incoming light is focused by a lens, typically filtered by a color filter array, and then detected by a 2D monochromatic detection array inside the camera. This measurement principle allows very-high-resolution imaging at high acquisition frequencies, but prohibits direct range measurements, as done with radar and LiDAR [7]. Deriving range information based on camera images can be done either using computer vision methods (e.g., based on object size) or by using stereo cameras and triangulation [8]. Additionally, velocity information can be calculated using, e.g., optical flow methods [9]. In particular, compared to radar, cameras perform less reliably under adverse weather conditions and at night. However, the camera is considered the most reliable perception sensor when it comes to object classification, lane detection, and traffic light recognition [10].

### 1.2. Virtual Testing of ADAS/AD Functions

Testing and validating ADAS functions based on camera systems is a major challenge for today’s SAE Level-2 vehicles. The effort to approve SAE Level-3+ vehicles, that will use cameras together with other perception sensors to support AD functions, will increase significantly since the responsibility of the environment perception is shifted from the driver to the system. Kalra and Paddock [11] demonstrated that fully autonomous vehicles would have to be driven hundreds of millions of kilometers and sometimes hundreds of billions of kilometers to demonstrate their reliability in terms of fatalities and injuries. Existing test fleets would take tens or hundreds of years to drive these kilometers. This proposes an impossible task since the demonstration of the vehicle performance needs to be completed prior to the release for consumer use. Hence, reducing the development effort for ADAS functions and eventually enabling AD functions demand the extension of conventional test methods, e.g., physical test drives, with simulations in virtual test environments [4,12], or mixed methods combining the both testing abstraction levels [13,14,15,16].

**Figure 1:**
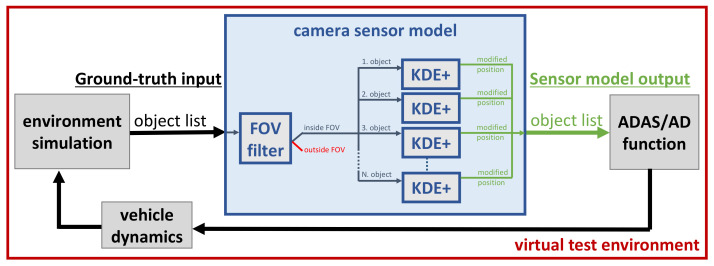
Camera sensor model at the object level embedded into a virtual test environment including environment simulation, ADAS/AD function, and vehicle dynamics.

In such a virtual test environment, a camera is simulated by a sensor model. The flowchart in Figure 1 illustrates the data flow of a virtual test environment for ADAS/AD functions, including the presented object-based camera model. An environment simulation, e.g., Vires VTD [17], IPG CarMaker [18], CARLA [19], AirSim [20], or aiSim [21], provides the test scenario including vehicles, pedestrians, etc., as the object list and forwards the true state of the environment (ground-truth) to the sensor model. The camera model reduces the ground-truth object list according to the field-of-view (FOV) of the camera and modifies the position estimation of the remaining objects according to the sensing capabilities of the respective camera. The camera model output is eventually fed into the ADAS/AD function under test. A promising approach to standardize the object list format for the interfaces between environment simulation, sensor model, and ADAS/AD function, called the Open Simulation Interface (OSI), is currently under development [22].

### 1.3. Previous Work on Automotive Camera Modeling

Schlager et al. [23] provided a comprehensive overview of models for automotive perception sensors and distinguished three categories of sensor models: low-fidelity (considering only geometrical aspects at the object level), medium-fidelity (including probabilistic and/or physical aspects at the object level), and high-fidelity (using rendering and ray tracing methods at the raw data level). Previous work on automotive camera modeling includes low-, medium-, and high-fidelity sensor models.

Low-fidelity sensor models that can simulate automotive cameras were given by Hanke et al. [24], Muckenhuber et al. [25], Schmidt et al. [26], Stolz and Nestlinger [27]. Hanke et al. [24], Schmidt et al. [26] suggested modifying the ground-truth object list sequentially in a number of modules, and each module shall represent a specific sensor characteristic or environmental condition. Stolz and Nestlinger [27] introduced a computationally efficient method to exclude all objects outside the sensor’s FOV. Muckenhuber et al. [25] presented a generic sensor model taking coverage, object-dependent fields of view, and false negative/false positive detections into account.

A medium-fidelity sensor model approach that can be used for automotive cameras was presented in Hirsenkorn et al. [28]. The sensor behavior was reproduced implicitly using conditional probability density functions based on sensor measurements and kernel density estimations.

Image rendering is typically performed embedded in the environment simulation. Therefore, high-fidelity camera models often use rendered images as the input and perform postprocessing steps in order to transform the ideal image into more realistic camera raw data. Examples for such high-fidelity camera models were given by Carlson et al. [29,30], Schneider and Saad [31], Wittpahl et al. [32]. Schneider and Saad [31] applied optical distortion, blur, and vignetting to modify the ideal image from the environment simulation. Wittpahl et al. [32] used point spread functions and neural networks to reduce the gap between synthetic and real images. Carlson et al. [29,30] presented an augmentation pipeline including chromatic aberration, blur, exposure, noise, and color temperature to simulate the image formation process and artifacts of a real camera.

### 1.4. Datasets for Automotive Camera Sensors

Sensor datasets help to understand the capabilities and limitations of perception sensors and, therefore, play an important role in assessing sensor performance and sensor modeling. In particular, sensor models based on probabilistic functions [28] or neural networks [30,32] require a representative labeled dataset to build realistic relationships between the ground-truth and sensor output.

Many sensor datasets are publicly available, and Kang et al. [33] provided an extensive overview of driving datasets with partial or full open access. Datasets are available including solely camera data [34,35,36,37,38], LiDAR and camera data [39,40,41], and camera, LiDAR, and radar data [42]. A common limitation of the above-listed datasets is the availability and quality of ground-truth data at the object level, in particular position estimations. Some datasets provide object labeling [42], but the labeling is typically based on the recording of the perception system and, hence, includes the measurement uncertainties of the perception system. To our knowledge, there is no larger dataset publicly available that includes both camera measurements at the object level and high-quality ground-truth position measurements utilizing highly accurate RTK assisted GPS localization both for the ego-vehicle and the target objects.

### 1.5. Scope of Work

This article deals with the development of an object-list-based sensor model, and the modeling approach was based on the work from Hirsenkorn et al. [28] with several extensions to improve the model performance and accuracy. For the evaluation of the modeling concept, a Mobileye 630 camera was chosen. The measurement data are provided at the object level and include the corresponding RTK-GPS position data of all attending vehicles as ground-truth information. The considered measurement campaign took place in 2020 on a Hungarian motorway [43].

### 1.6. Structure of the Article

Section 2 introduces a sensor model based on object lists; the kernel density approach is summarized, and the sensor model development is described in detail. Section 3 provides a description of the measurement campaign, including the measurement hardware specifications and a detailed scenario description. Section 4 evaluates the sensor model’s performance. Section 5 completes the paper with a summary and conclusion and gives an outlook on future work.

## 2. Object-List-Based Sensor Model

The output of many different perception sensor types, especially when developed for the automotive industry, is a so-called object list. This means that, e.g., an automotive camera processes the recorded image internally and provides as the output a list of detected objects with position estimations. Based on this level of information, a sensor model is developed by utilizing a statistical method, called kernel density estimation theory. For more details on the applied methods, see, for example, Parzen [44], Turlach [45].

### 2.1. Kernel Density Estimation: A Short Introduction

Kernel density estimation methods (KDE) are wide spread and well-known for approximating the distribution of given measurements or a dataset. The following section is a short and simplified summary of parts from Parzen [44] and Turlach [45]. One of the major benefits of this technique is that no knowledge of the underlying distribution of the measurements is required. This nonparametric nature guarantees that the shape of the distribution will be automatically learned from the data; see Hirsenkorn et al. [28].

It is assumed that there are different measurements $x_{1},x_{2},\ldots ,x_{n}$ corresponding to each object. Every measurement is assigned to a kernel function *K*, and based on these functions, the distribution of the whole measurements can be approximated by:

(1) $$\begin{array}{c} f\left(x\right):=\frac{1}{nh}\sum_{i=1}^{n}K\left(\frac{x-x_{i}}{h}\right), \end{array}$$

where *n* represents the number of measurements and *h* the bandwidth. The greater *h* is, the smoother *f* will be, and the smaller *h* is, the less smooth *f* will be. This respectively corresponds to the underfitting and overfitting of *f*. There are many different choices of the kernel function *K*. The most popular ones are, for example, the Gaussian kernel:

(2) $$\begin{array}{c} K_{G}\left(t\right):=\frac{1}{2\sqrt{\pi }}exp\left(-0.5t^{2}\right), \end{array}$$

the uniform kernel:

(3) $$\begin{array}{c} K_{U}\left(t\right):=\left\{\begin{array}{c} 0.5\;\mathrm{for}\;|t|<=1\\ 0\;\mathrm{else} \end{array}\right. \end{array}$$

or the triangle kernel:

(4) $$\begin{array}{c} K_{T}\left(t\right):=\left\{\begin{array}{c} 1-|t|\;\mathrm{for}\;|t|<=1\\ 0\;\mathrm{else}. \end{array}\right. \end{array}$$

**Figure 2:**
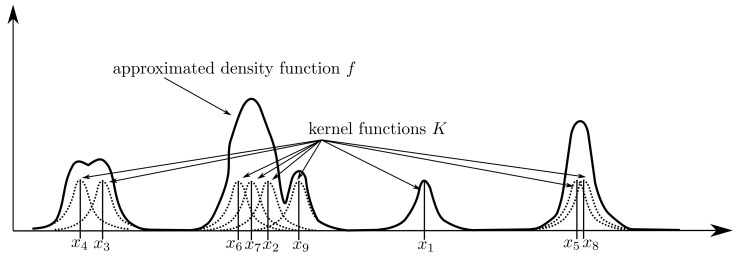
Schematic representation of approximating a density function from measurements by utilizing kernel density estimation methods.

Many more kernels exist (see, e.g., Turlach [45]), but all used kernels have to be symmetric, i.e., K(t)=K(−t), and need to fulfil $\int_{-\infty }^{\infty }K\left(\tau \right)d\tau =1$. This is required to guarantee that *f* is a density function. The construction of the approximated density function *f* in (1) from measurements is schematically illustrated in Figure 2.

### 2.2. Sensor Model Development

The object-list-based sensor model expects as an input an object list containing the *x* and *y* positions of the detected objects, these inputs are typically provided by an environment simulation. In the first step of the model, it has to be determined which objects are inside the field of view of the sensor. This is performed by a field of view (FOV) filter. The FOV is described by a sector of a circle defined through an angle and radius; the objects that are outside the FOV are removed, and the remaining objects are given as an input to the statistical KDE+ sensor model, which produces a modified *x* and *y* position for every object. These modified positions are gathered in an object list, which represents the output of the sensor model. The structure of the model is illustrated in the blue box of Figure 1. The FOV filter is introduced and explained in Muckenhuber et al. [46]. The statistical KDE+ model is introduced, explained, and discussed in the following.

The development of the KDE+ sensor model was based on the comparison of measured sensor data, always denoted with the subscript ${}_{sens}$, and the corresponding ground-truth values, denoted with the subscript ${}_{GT}$. For easier understanding of the model development, there are three different stages of the KDE+ sensor model: (i) polar coordinate model, (ii) inertia model, and (iii) extension with distance-based correction. The polar coordinate model is evolved into the inertia model, which is then enhanced by the distance-based correction.

**Figure 3:**
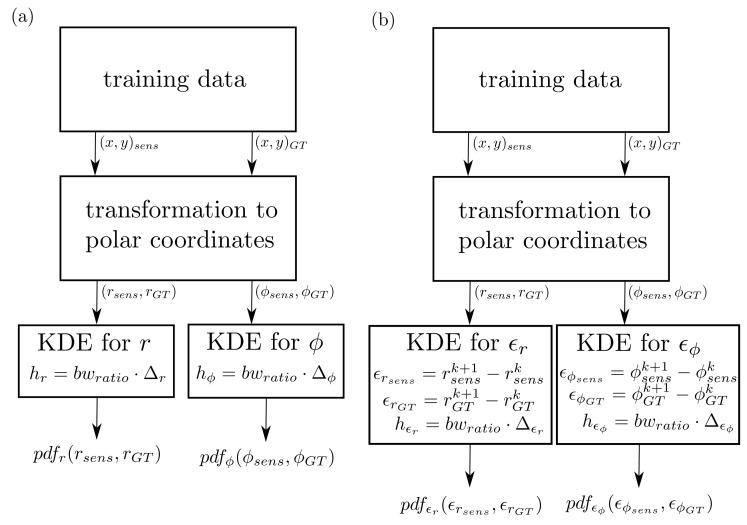
Workflow of developing the polar coordinate sensor model (**a**) and the inertia sensor model (**b**).

**(i) Polar coordinate model**: The development of the polar coordinate model is illustrated in the left part of Figure 3. The training data represent object lists with the *x* and *y* position recorded by the sensor denoted as ${\left(x,y\right)}_{sens}$ and the corresponding ground-truth values as ${\left(x,y\right)}_{GT}$. The choice of the training data is of high importance for the quality of the sensor model. By quality, it is meant that the effects one wants to simulate with the sensor model have to be captured in the training data, e.g., if the sensor model is used for observing cut-in scenarios, then in the training data, cut-in scenarios should be present as well. The required range of the sensor model has to be sufficiently covered by the training data. As a first step, the Cartesian coordinates are transformed to polar coordinates ${\left(r,\phi \right)}_{sens}$ and ${\left(r,\phi \right)}_{GT}$, by applying:

(5) $$\begin{array}{cc} r & =\sqrt{x^{2}+y^{2}}, \end{array}$$

(6) $$\begin{array}{cc} \phi & =arc\;tan(\frac{y}{x}). \end{array}$$

These values are required for constructing a probability density function (pdf) by the kernel density estimation theory for the distance *r* and the angle ϕ, respectively. The transformation from Cartesian to polar coordinates is applied because the detection error of the sensor depends mainly on the distance from the object and not the specific *x* and *y* coordinates.

For the construction of the two-dimensional KDE for the distance *r* the quantities $r_{sens}$ and $r_{GT}$ are utilized; this means the pdf is a two-dimensional function of $r_{sens}$ and $r_{GT}$. The required bandwidth:

(7) $$\begin{array}{c} h_{r}=bw_{ratio}\Delta_{r} \end{array}$$

is computed by a user-defined parameter $bw_{ratio}$, which is typically in the range of [0.01,0.0001]. The benefit of using this approach is that only one parameter has to be chosen, and it is not influenced by a scaling of the trainings data, e.g., changing units. This means the same $bw_{ratio}$ will work for many different scenarios in a satisfying way. The range of the distance training data is defined as:

(8) $$\begin{array}{c} \Delta_{r}=max\left(max\left(r_{sens}\right),max\left(r_{GT}\right)\right)-min\left(min\left(r_{sens}\right),\min\left(r_{GT}\right)\right). \end{array}$$

The sensor model itself is then represented by the two-dimensional probability density function $pdf_{r}\left(r_{sens},r_{GT}\right)$. This is typically saved as a two-dimensional array with a fixed size, where the first dimension represents the range of the measured sensor data and the second dimension stands for the ground-truth values. For the angle ϕ, the construction of $pdf_{\phi }\left(\phi_{sens},\phi_{GT}\right)$ is analogous to the construction of $pdf_{r}\left(r_{sens},r_{GT}\right)$.

**(ii) Inertia model**: The difference between the inertia and the polar coordinate model is that the pdf is constructed for the difference of two consecutive positions instead of the absolute positions, as is illustrated in the right part of Figure 3. The input for the two-dimensional KDE is defined as:

(9) $$\begin{array}{cc} \epsilon_{r_{sens}}\left[k\right] & :=r_{sens}^{k+1}-r_{sens}^{k}, \end{array}$$

(10) $$\begin{array}{cc} \epsilon_{r_{GT}}\left[k\right] & :=r_{GT}^{k+1}-r_{GT}^{k}, \end{array}$$

where k=1,…,n−1 and *n* denotes the number of samples in the dataset. The bandwidth and the range have to be appropriately adapted, leading to the two-dimensional $pdf_{\epsilon_{r}}\left(\epsilon_{r_{sens}},\epsilon_{r_{GT}}\right)$. As above, for the angle ϕ, the construction of $pdf_{\epsilon_{\phi }}\left(\epsilon_{\phi_{sens}},\epsilon_{\phi_{GT}}\right)$ works analogously.

**Figure 4:**
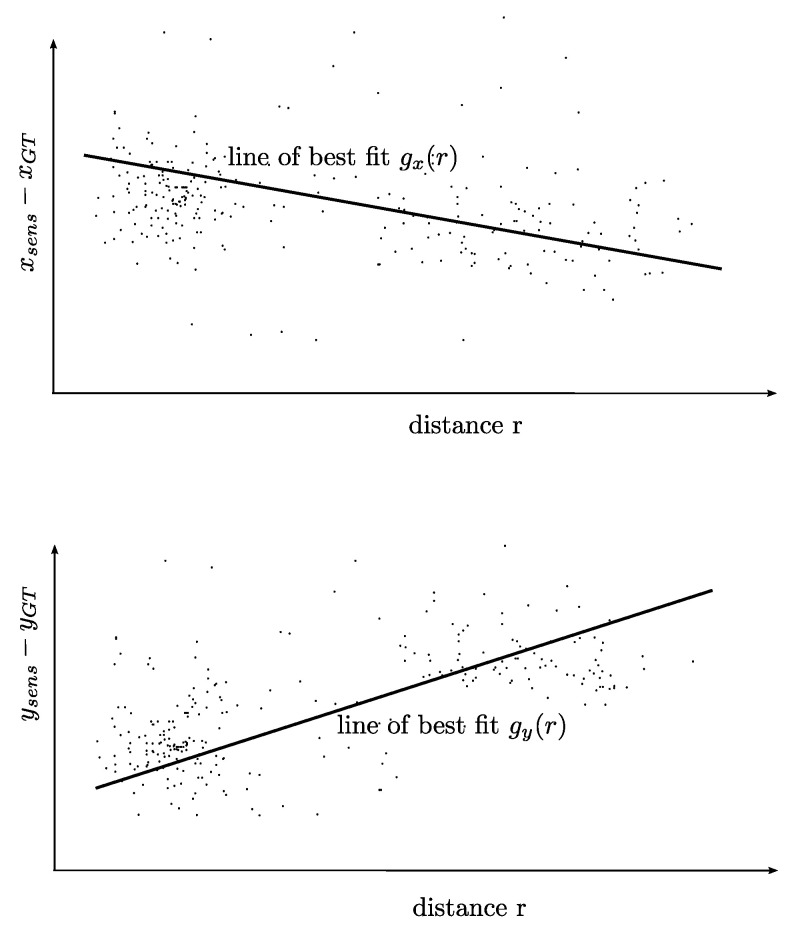
Schematic illustration of the scatterplot and the line of the best fit, which represents the distance-based correction.

**(iii) Extension with distance-based correction**: An analysis of the training data according to the dependency of the difference $x_{sens}-x_{GT}$ from the distance leads to the regression model, which is the basis of the distance-based correction extension of the sensor model. In the upper part of Figure 4, the scatterplot representing the training data in the *x* direction and the regression model of first order $g_{x}\left(r\right)$, i.e., the line of the best fit, are schematically depicted. The line of the best fit $g_{x}\left(r\right)$ represents the distance-based correction, i.e., for a given distance *r*, the value $g_{x}\left(r\right)$ is added to the output of the sensor model in the *x* direction. For the *y* direction, it works analogously.

Summarizing, this means that the KDE+ model, the inertia model with the distance-based correction, works as follows: The input is the *x* and *y* position of an object in the FOV of the sensor. The input is transformed to polar coordinates *r* and ϕ. The next step is calculating the difference between the current and the last sample for the distance and angle, respectively, leading to $\epsilon_{r}$ and $\epsilon_{\phi }$. Inserting these two values in the two-dimensional pdfs at the position of the ground-truth values, the one-dimensional pdfs:

(11) $$\begin{array}{c} pdf_{\epsilon_{r}}\left(\cdot ,\epsilon_{r}\right) \end{array}$$

and:

(12) $$\begin{array}{c} pdf_{\epsilon_{\phi }}\left(\cdot ,\epsilon_{\phi }\right) \end{array}$$

are computed. The output of the sensor model is then computed by choosing randomly a value of each one-dimensional pdf (11) and (12) and then adding it to the input *x* and *y* coordinates, resulting in the modified position of the object.

## 3. Validation Data: Measurement Campaign

A significant element of ADAS/AD function development is the collection of measurement data, which are typically utilized in both training and validating the AI-based perception algorithms (e.g., semantic segmentation, object detection, etc.) and the control algorithms utilizing them. In this sense, the importance of validated models for ADAS/AD function development is described in Section 1 as a motivation. Here, we describe particular validation test data that were utilized as ground-truth information, which enabled the development of the statistical object-list-based medium-fidelity model described in Section 2.

### 3.1. Campaign Description

Collecting data for ADAS/AD function development and validation is not a straightforward task and typically takes a great deal of time and effort. It is a general problem that high-precision ground-truth data are hardly available as part of the validation tests. Validation is a necessary step to determine the correlation of the model with the real world. This is of utmost importance to determine (a) how well the model fits the real world, (b) what error margins are to be expect due to the assumptions made while modeling, and (c) how it helps to create an understanding of the significance of the model and its limitations. In such validation measurements, the test data typically consist of only the ego-vehicle behavior, and external measurements of the scenario and the behavior of the other dynamic objects (e.g., other vehicles) are usually unavailable.

**Figure 5:**
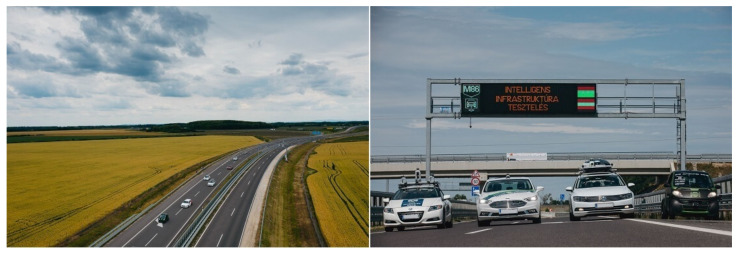
Austro-Hungarian Test Campaign conducted in June 2020.

In 2018, Hungary, Slovenia, and Austria signed a Memorandum of Understanding (MoU) as a cross-border cooperation agreement at the ministerial level to support the development and testing of electric, connected, and self-driving automotive technologies [47]. Based on this agreement, a bilateral call for exploratory projects was issued to prepare transnational R&D projects between Austria and Hungary (see the Acknowledgment Section for the project details). As a dedicated activity of this exploratory phase, a measurement campaign was carried out on a real-world motorway stretch of Hungary with the participation of international industrial and academic partners (see Figure 5). The measurement campaign generated ground-truth sensor data from both the vehicle and infrastructure perspectives, which proved to be extremely useful for future automotive research and development activities, in particular in the automated vehicle domain, due to the availability of the ground-truth information for static and dynamic content [43]. All the vehicles used in this testing campaign were equipped with high-accuracy differential Global Navigation Satellite Systems (GNSSs) for localization, each of which were calibrated for accurate positioning information.

This calibration process was conducted on the ZalaZONE proving ground before the testing campaign on the closed M86 Csorna High-Way test section. For the calibration, a specific position on the ZalaZONE proving ground was selected and used as a high-precision reference point. Each test vehicle was placed exactly at this position, and the onboard GNSS measurements with RTK corrections from the same mobile base station were taken. Then, utilizing the mounting position of the antennas, as well as the outer dimensions of the vehicle, the accuracy information was obtained, and a calibration compared to the reference point was performed.

**Figure 6:**
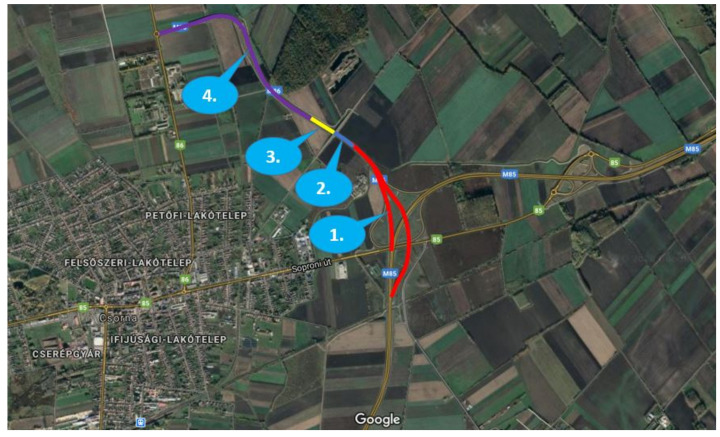
Road sections of the test site (3.5 km in all) located near Csorna City (Hungary) on Route E65 (GNSS coordinates: 47.625778, 17.270162).

The measurement campaign was carried out on a highway section near the town of Csorna, in the northwestern part of Hungary, which is located at the crossing of two main regional highway sections, M85 and M86, seen in Figure 6. There are four sections of the road with different characteristics, where mainly road sections 1 and 2 were used in the scope of the measurement campaign. With reference to Figure 6, the features of these sections are as follows:

- Road section 1. Interchange area (red): The two carriageways have different horizontal and vertical alignment, while leaving the M85-M86 interchange. In this section, two 3.50 m-wide lanes are available for the through traffic, and there are additional accelerating/decelerating lanes linked to junction ramps;
- Road section 2. Open highway (blue): A common, approximately 300 m-long dual-carriageway section with two 3.50 m-wide traffic lanes and a 3.00 m-wide hard shoulder on both sides.

A total of 13 different vehicles participated in the test campaign, which comprised dissimilar passenger cars and two trucks, one also including a trailer. Different numbers of vehicles took part in various test drives depending on the test scenario. All of the test vehicles had calibrated high-accuracy GPSs, along with additional onboard sensors [43]. The setup of the test vehicle from Virtual Vehicle Research GmbH (VIF) is described in detail next.

### 3.2. Test Setup and Measurement Hardware

**Figure 7:**
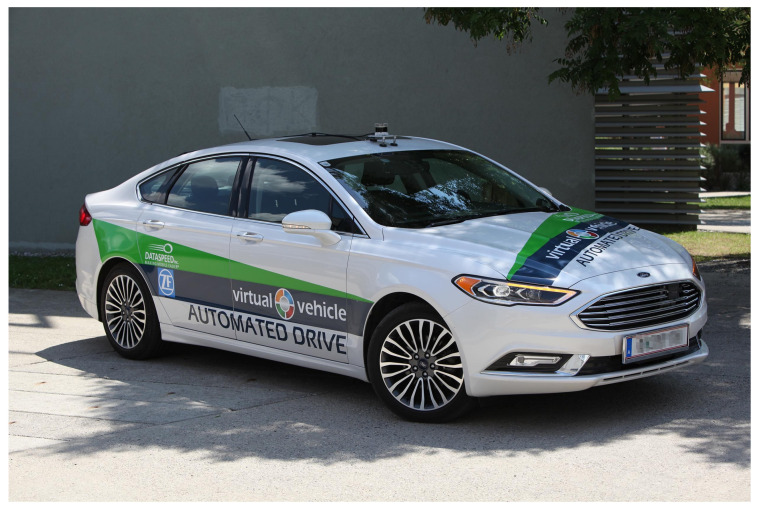
VIF’s Automated Drive Demonstrator (ADD) vehicle.

Virtual Vehicle Research GmbH (VIF) is a research organization that is actively working on all areas of model-based vehicle development, in particular including automated driving system solutions. Of special interest is the development of tools and methodologies that can aid in the Scenario based validation and verification of ADAS/AD systems at various abstraction levels spanning simulation-only and real-life testing. With this motivation and background, VIF joined the measurement campaign with one of its generic Automated Drive Demonstrator (ADD) vehicles. A Ford Fusion Hybrid MY2017 (see Figure 7) was the vehicle used for this purpose, which is equipped with several additional sensors and computational hardware, as well as custom software components.

The ADD vehicle sensor setup can be modified depending on the measurement or the use case requirements. A previous example for this is from the EU/ECSEL project PRYSTINE, where robust multisensor fusion using additional sensor modalities was developed and demonstrated on an automated valet parking use case [48]. Another similar implementation was performed in the scope of the EU project INFRAMIX, where the focus was infrastructure-assisted ADAS implementations and C-ITS integration with the ADD vehicle [15].

**Figure 8:**
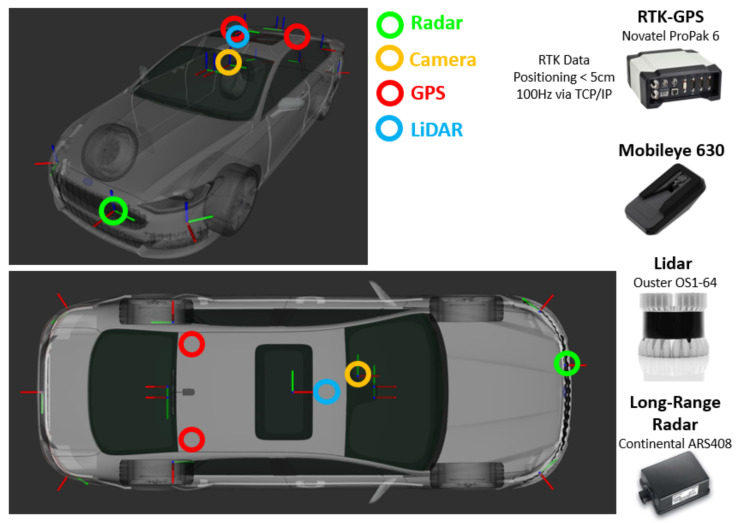
VIF’s ADD vehicle sensor setup and the corresponding mounting positions.

To support the aim of this measurement campaign, the VIF ADD vehicle was equipped with a high-accuracy dual-antenna DGPS to provide ground-truth location information. A Novatel ProPak6 RTK-GPS receiver was utilized for the measurement of the precise position supported by a TCP/IP-based RTK correction service providing sustained centimeter-level accuracy. Additionally, the VIF ADD vehicle also logged other sensor data relevant to the perception algorithm’s development and validation purposes. These sensors specifically included a Mobileye 630 series intelligent camera, a Continental ARS408 long-range radar (https://conti-engineering.com/components/ars-408/, accessed on 15 September 2021), and an Ouster OS1-64 LiDAR sensor (https://ouster.com/products/scanning-lidar/os1-sensor/, accessed on 15 September 2021). Figure 8 shows the mounting positions of the perception sensors. For the data acquisition, an ROS-based AUTOWARE.AI (https://www.autoware.ai/, accessed on 15 September 2021) software stack running on an Ubuntu X86-PC was utilized to log the data in rosbag format.

### 3.3. Scenario Descriptions

In this section, the relevant scenarios utilized for the development of the Mobileye camera model are introduced. The inspiration for these scenarios partially came from a recent and extensive UN approval document, namely Regulation No. 157 (ECE/TRANS/WP.29/2020/81) on Automated Lane Keeping Systems (ALKS), where a cut-in scenario is described. The choice of other scenarios stemmed from sensor separability and occlusion tests with the purpose of the development and validation of sensor models. The measurements performed were conducted exclusively with manually driven vehicles, since the focus was the gathering of ground-truth sensor data. Therefore, the safety of the driving functions or the related standard compliance was not considered. The driving safety was ensured by the test drivers.

The measurements of VIF Scenarios 1–3 were performed on 24 June 2020 at different times of the day; the measurement for the C-ITS Scenario was performed on 25 June.

#### 3.3.1. Sensor Scenario 1 (Cut-In)

**Figure 9:**
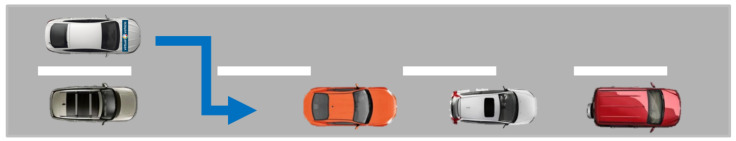
M86 Test Scenario 1 with the ego-vehicle cutting in.

In this scenario, 5 vehicles are moving at a constant speed (approximately 10–20 km/h), as depicted in Figure 9, on two lanes. The ego-vehicle is driving on the left lane before it cuts in suddenly to the free space in front of the last vehicle. Data measurement for this scenario was performed during the daytime (evening, 7 p.m.) and good weather conditions (sunny with a few scattered clouds).

#### 3.3.2. Sensor Scenario 2 (Occlusion)

**Figure 10:**
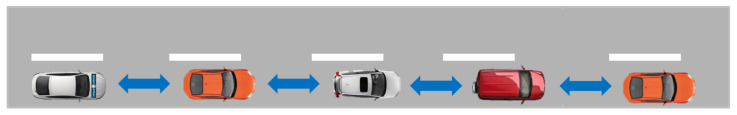
M86 Test Scenario 2 with the ego-vehicle in the rear position.

In this scenario, a convoy of five vehicles is moving at a constant speed (approximately 10–20 km/h) according to Figure 10, while the distance between the vehicles is varied equally. The ego-vehicle is in the last position behind the convoy. The target distances between each vehicle were set consecutively as 1 m, 5 m, 10 m, 30 m, and 50 m. Data measurement for this scenario was performed during the daytime (evening, 9 p.m.) and good weather conditions (sunny with a few scattered clouds).

#### 3.3.3. Sensor Scenario 3 (Separability)

**Figure 11:**
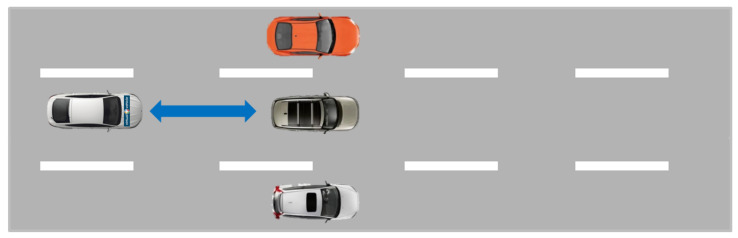
M86 Test Scenario 3 with the ego-vehicle in the rear position.

In this scenario, three vehicles are next to each other, as depicted in Figure 11, with the ego-vehicle placed behind, in the middle lane. The three target vehicles drive slowly away (around 10–20 km/h), while the ego-vehicle stays stationary or vice versa. Data measurement for this scenario was performed during the daytime (afternoon, 4 p.m.), under good weather (sunny with some scattered clouds) and lighting conditions.

#### 3.3.4. C-ITS Scenario 1 (Variable Speed Limits)

**Figure 12:**
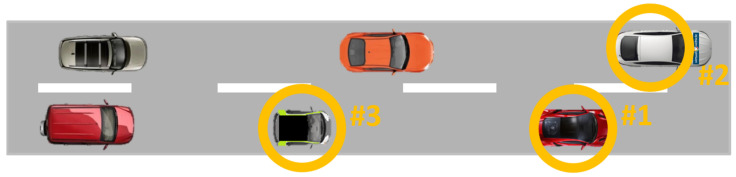
M86 Test Scenario 4 with the ego-vehicle in the lead position.

In this scenario, vehicles are moving at a constant speed of 40 km/h according to Figure 12, where Car#2 represents the ego-vehicle’s starting position. The variable message sign (VMS) indicates a reduced 30 km/h speed limit. In the first run, only Car#1 respects the new speed limit, while the others ignore the message and drive at the original speed. In the second run, two vehicles (Car#1 and Car#2) respect the new speed limit, whereas in the third run, three vehicles (Car#1, Car#2, Car#3) respect the new speed recommendation. The data measurement for this scenario was performed during the daytime (10 a.m.) under good, but partly cloudy weather conditions.

## 4. Sensor Model Evaluation

Combining the object-list-based sensor model from Section 2 with the data from the measurement campaign from Section 3 leads to a model of the Mobileye 630 camera. In the following, the evaluation of this camera sensor model is discussed.

### 4.1. Sensor Models

As stated in Section 2, there are three different stages of the sensor model: (i) the polar coordinate model, (ii) the inertia model, and (iii) the inertia model extended by the distance-based correction. These three models are described in the following. It should be mentioned that instead of the probabilistic distribution functions (pdfs), the cumulative distribution functions (cdfs) are depicted because the important properties are better recognizable. The cdf is the integral of the pdf, which means both functions contain exactly the same information: nothing is added or lost.

**Figure 13:**
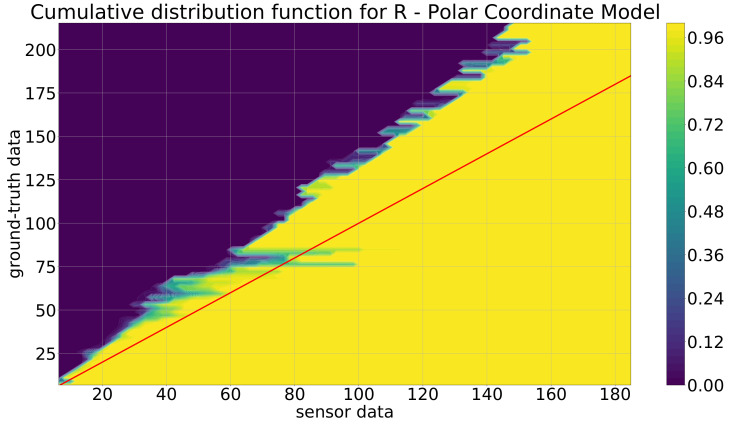
Cumulative distribution function for the polar coordinate model for the distance *r* (m).

**Figure 14:**
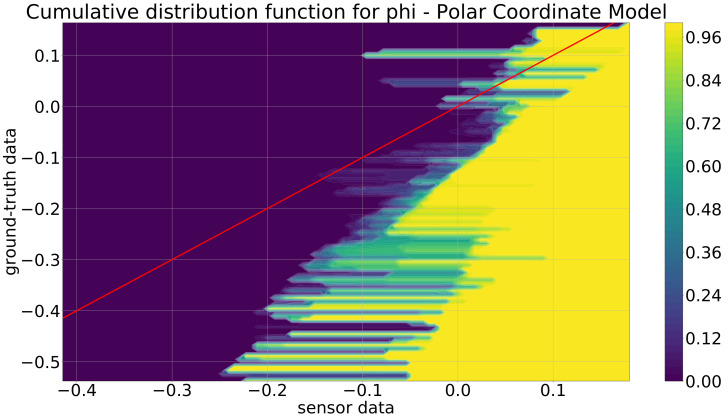
Cumulative distribution function for the polar coordinate model for the angle ϕ (deg).

As the polar coordinate model is the first stage of the model development, it is discussed first. At this first abstraction level, the user-defined parameter $bw_{ratio}$ was chosen as $bw_{ratio}=0.001$ for all the KDE+ models, and as kernel functions, the Gaussian kernel from Equation (2) was selected. The value of $bw_{ratio}=0.001$ was found as a good choice by a small number of comparisons of different values in the range [0.01,0.0001]. In Figure 13 and Figure 14 are the cdfs of the KDE+ model depicted, which were constructed by utilizing the training data described in the previous section. For the cdf for the distance *r* (Figure 13), the red line indicates where the sensor and ground-truth distance values are equal. The increase of the cdf, i.e., the fast change from small values ≈0 (violet) to high values ≈1 (yellow), is always above the red line, meaning that the Mobileye camera always detects the objects that are too close, e.g., for the part for the ground-truth data at ≈100 m, the increase of the cdf is at the position of ≈80 m in the sensor data. Additionally, this difference increases nearly linearly by increasing the ground-truth or sensor values. This leads to the conclusion that the Mobileye camera always detects the objects that are too close, and for an increasing distance, this effect increases as well. In Figure 14, the cdf for the angle ϕ is depicted. Here, a similar trend is not observable since the rise is beneath the red line for negative values and above for positive ones.

**Figure 15:**
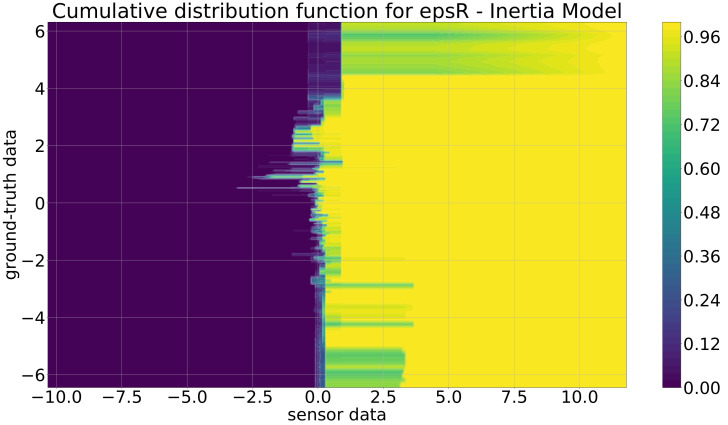
Cumulative distribution function for the inertia model for $\epsilon_{r}$ (m).

**Figure 16:**
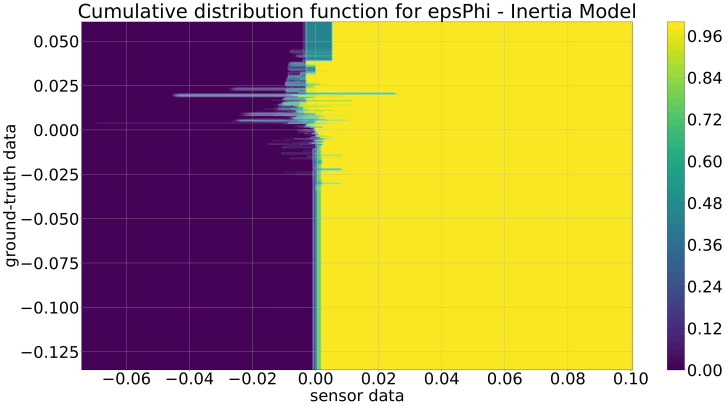
Cumulative distribution function for the inertia model for $\epsilon_{\phi }$ (deg).

In Figure 15, the cdf for the inertia model is depicted. Here, it is obvious that in contrast to the cdfs from the polar coordinate model, the shape of the rise of this cdf is completely different. For ground-truth values in [−6,−2] and [4,6], the rise is nearly independent of the ground-truth value. This comes from the fact that these large ground-truth values are really rare in the training data, and so, these parts are not really valid. However, this is no problem, as typically, the position from one sample to the other will not vary with such a high speed, because, e.g., a ground-truth value of −5 m and a typical sampling rate of 0.1 s mean that an object is approaching the ego-vehicle with 50 m/s = 180 km/h. In the more relevant part in [−2,4], it is obvious that the increase of the cdf fluctuates and is mostly negative for the sensor data. This means that, typically, the sensor distance measurement is lower than the ground-truth data, which is a similar effect as the one observed in the polar coordinate model. For the cdf of the angle in Figure 16, similar effects can be seen.

**Figure 17:**
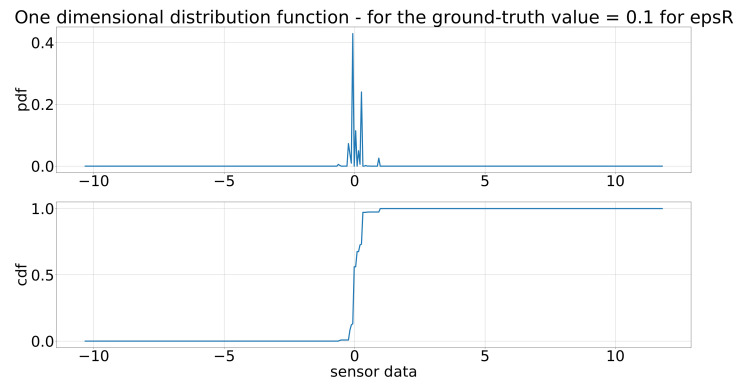
One-dimensional distribution functions of the inertia model for $\epsilon_{R}$ for a value of 0.1 (m).

**Figure 18:**
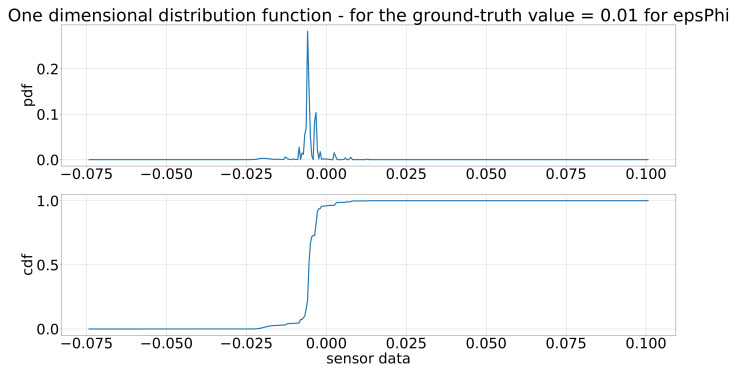
One-dimensional distribution functions of the inertia model for $\epsilon_{\phi }$ for a value of 0.01 (deg).

As described at the end of Section 2.2, the two-dimensional pdf and cdf were evaluated for specific positions in place of the ground-truth values, leading to a one-dimensional pdf and cdf. These one-dimensional pdf and cdf are depicted in Figure 17 for the distance of the inertia model for a value of 0.1 m and in Figure 18 for the angle of the inertia model. As the input in every step, the ground-truth values, of the sensor model changes, the two-dimensional distributions functions have to evaluated for a different ground-truth value, leading to different one-dimensional distribution functions in every step. These one-dimensional pdf and cdf are the basis of generating the output of the sensor model in every step.

**Figure 19:**
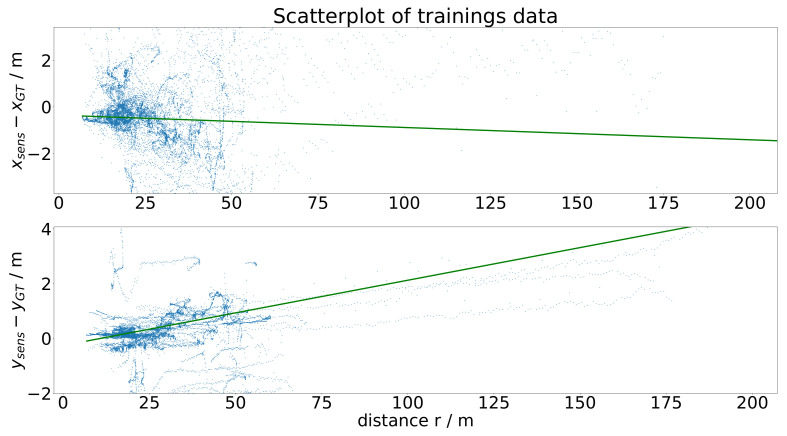
Scatterplot and the line of the best fit, as a basis for the distance-based correction.

The third step of the sensor model is the extension with the distance-based correction. This correction is computed with the analysis of the dependency of the gap between sensor and measurement data from the distance of the object, as depicted in Figure 19. The green line in the figure denotes the line of the best fit, which is the linear regression model utilized for the distance-based correction. This means that for increasing distance, we see that $x_{sens}-x_{GT}$ is clearly negative and $y_{sens}-y_{GT}$ is clearly positive, and both starting from a nearly zero gap for small distances. This fits the previously observed effects perfectly, as the error between the sensor and ground-truth data increases with the distance from the object.

### 4.2. Results

In this section, the results of the three different stages of the sensor model of the Mobileye camera are evaluated and discussed. The test data for this evaluation were from a detected object from the measurement campaign in Section 3 that is excluded from the training data. The trainings data were the scenarios described in Section 3.3, and the test data consisted of one detected object of a scenario, which was chosen randomly. Therefore, a comparison to the ground-truth positions was possible, leading to a high-quality evaluation. In this case, the ground-truth RTK-GPS position data were the input to the sensor model, and the measured data were the reference data for the sensor model output.

**Figure 20:**
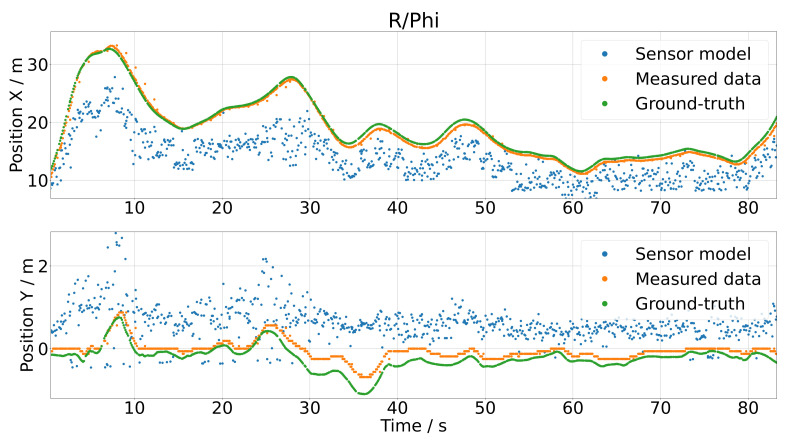
Results of the polar coordinate model by using the test data as the input.

**Figure 21:**
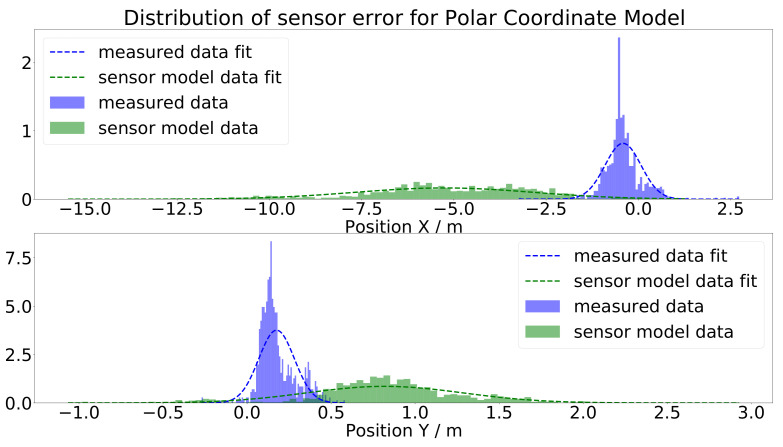
Histogram and Gaussian fit of the gap between the measured, respectively simulated, sensor data and the ground-truth values for the polar coordinate model.

In Figure 20, the *x* and *y* positions of the polar coordinate model are compared to the real measurements (orange dots) and the ground-truth data (green dots). The output of the sensor model (blue dots) was not satisfying, as there were huge gaps between the measured data and the output of the sensor model. Especially for the *y* position, the output of the model nearly appeared as random noise. In Figure 21, the histograms of the measured and simulated sensor errors are depicted, i.e., the difference of the sensor output and the ground-truth data (green) and the difference of the measured sensor data and the ground-truth data (blue) are depicted. For a satisfying model, both histograms should coincide, as the histograms describe the distribution of the gap between the ground-truth and measured simulated data. For the polar coordinate model, this is obviously not the case.

**Figure 22:**
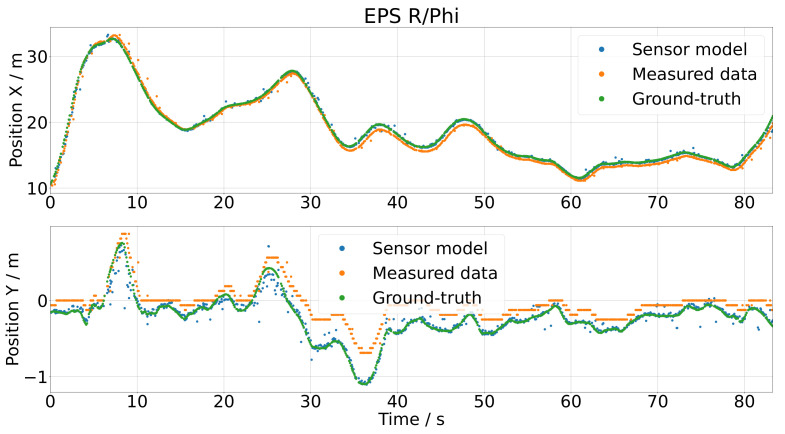
Results of the inertia model by using the test data as the input.

**Figure 23:**
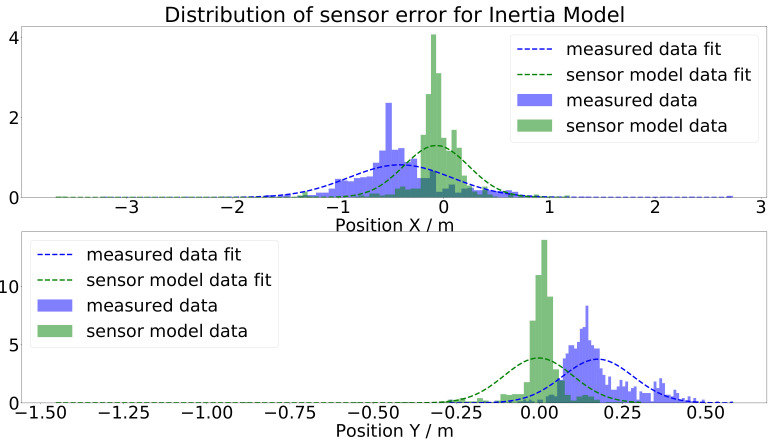
Histogram and Gaussian fit of the gap between the measured, respectively simulated, sensor data and the ground-truth values for the inertia model.

Applying the inertia model on the same test data led to the results depicted in Figure 22. The results were very different from the polar coordinate model. The scattering of the sensor model output was satisfying, the only remaining issue being that the results were too close to the ground-truth data. The fact that the scattering looks realistic can be more precisely verified by plotting the histograms of the sensor errors in Figure 23. There, it is easy to see that the shape of the histogram looks very similar for the *x* and *y* position; they are only shifted. This means that the scattering or the natural deviations of the measured camera data were modeled with a satisfying accuracy by the inertia model.

**Figure 24:**
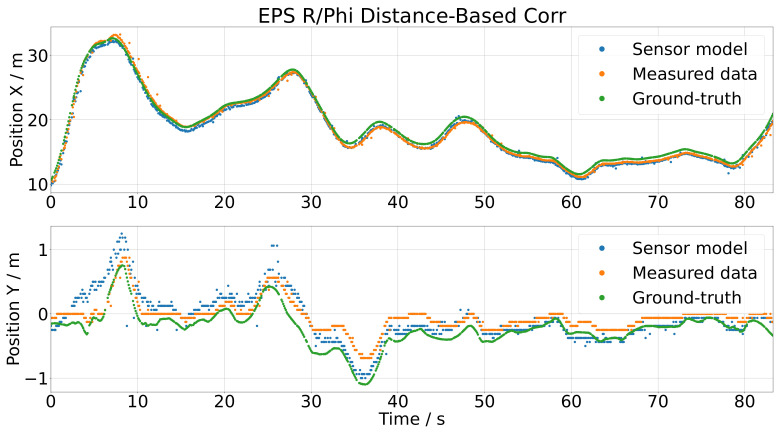
Results of the inertia model extended by the distance-based correction by using the test data as the input.

**Figure 25:**
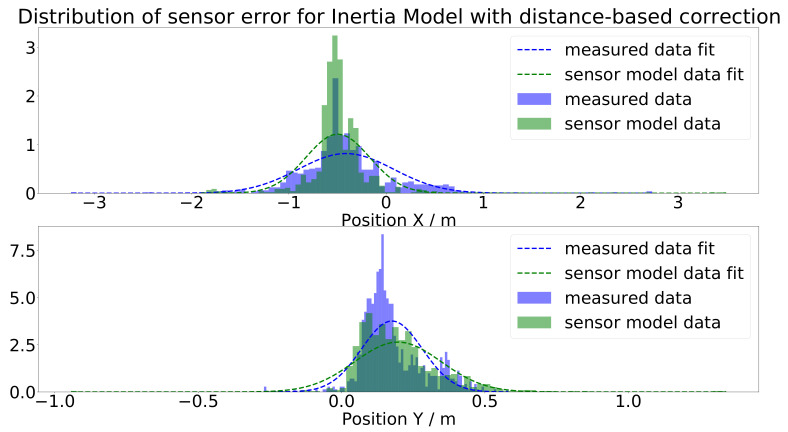
Histogram and Gaussian fit of the gap between the measured, respectively simulated, sensor data and the ground-truth values for the inertia model extended with the distance-based correction.

The inertia model extended by the distance-based correction is the final version of the sensor model, and as shown in Figure 24, it generated the best results. To measure the accuracy of the sensor model, a pointwise error measure was utilized by comparing the measured data and the output of the sensor model. To compare the position error in the *x* and *y* direction reasonably, they were each normalized by its range: for *x*, the range was 22.875 m, and for the *y* direction, it was 1.563 m. This led to a position error in the *x* direction of $err_{x}=1.57\%$ and in the *y* direction of $err_{y}=9.6\%$. The scattering of the sensor output was satisfying and really close to the measured data. This is additionally shown in the histograms in Figure 25 as the blue and green histograms, and also, the fitted distributions nearly coincided. The extension with the distance-based correction shifted the histogram of the simulated sensor error correctly, as one can see by comparing Figure 23 and Figure 25.

## 5. Summary and Conclusions

This paper focused on a modeling approach for object-list-based sensor models. The concept of kernel density estimation theory was combined with regression theory. The development of this sensor model required three stages, starting with the polar coordinate model, where every position of an object was treated independently. This approach was enhanced by considering the continuity or inertia of objects, i.e., objects cannot appear or disappear spontaneously, resulting in the so-called inertia model. Extending this modeling approach by a distance-based correction based on linear regression led to the final stage of the sensor model the inertia model with distance-based correction.

As the kernel density and also the linear regression approaches are statistical and data-driven methods, the sensor models require appropriate training data. Therefore, high-quality measurement data from a measurement campaign on the Hungarian highway were utilized. All involved dynamic objects in the measurement campaign were equipped with an RTK DGPS, meaning that for every object, accurate ground-truth measurements were available. This is a major benefit when comparing this dataset to other available open-source datasets. The RTK DGPS data allowed training the models based on the difference of the measured data from the Mobileye 630 camera and the ground-truth measurements.

To evaluate the presented modeling approach, the Mobileye 630 camera was chosen, since the utilized measurement data were recorded with this camera. For a profound evaluation, the test data were chosen carefully, and a part of the measurement data was used to test the sensor model. The chosen test data were not used to train the sensor model, as this would disturb the evaluation significantly. Based on the evaluation with the test data, pointwise position errors of 9.60% in the lateral and 1.57% in the longitudinal direction were found. The model was able to represent the position estimation fluctuations of the Mobileye camera very well.

Future work will deal with an analysis of the influence of the bandwidth of the kernel density approach. Of additionally interest is generalizing this modeling concept for the development of a lane-marking model or taking other signals such as the velocities of an object into account.

Another ongoing research topic is the creation of realistic training data based on simulations. The currently available datasets are typically recorded in physical test drives, with the advantage of representing real sensor measurements in real-life scenarios. However, the challenges of creating training data in physical test drives are (i) the great effort and cost connected with real physical test drives and (ii) the recording of ground-truth data. In the near future, it might be possible to produce realistic and representative datasets based on very detailed environment simulations combined with high-fidelity sensor models or sensor stimulation. This could potentially solve the ground-truth data issue and allow us to create a very large amount of training data, which would not be feasible with physical test drives.
